# Introduction of shared electronic records: multi-site case study using diffusion of innovation theory

**DOI:** 10.1136/bmj.a1786

**Published:** 2008-10-23

**Authors:** Trisha Greenhalgh, Katja Stramer, Tanja Bratan, Emma Byrne, Yara Mohammad, Jill Russell

**Affiliations:** 1 Research Department of Primary Care and Population Health, University College London, Highgate Hill, London N19 5LW

## Abstract

**Objective** To explore the introduction of a centrally stored, shared electronic patient record (the summary care record (SCR)) in England and draw wider lessons about the implementation of large scale information technology projects in health care.

**Design** Multi-site, mixed method case study applying utilisation focused evaluation.

**Setting** Four early adopter sites for the SCR in England—three in urban areas of relative socioeconomic deprivation and the fourth in a relatively affluent rural area.

**Data sources and analysis** Data included 250 staff interviews, 1500 hours of ethnographic observation, interviews and focus groups with 170 patients and carers, 2500 pages of correspondence and documentary evidence, and incorporation of relevant surveys and statistics produced by others. These were analysed by using a thematic approach drawing on (and extending) a theoretical model of complex change developed in a previous systematic review.

**Main findings** The mixed fortunes of the SCR programme in its first year were largely explained by eight interacting influences. The first was the SCR’s material properties (especially technical immaturity and lack of interoperability) and attributes (especially the extent to which potential adopters believed the benefits outweighed the risks). The second was adopters’ concerns (especially about workload and the ethicality of sharing “confidential” information on an implied consent model). The third influence was interpersonal influence (for example, opinion leaders, champions, facilitators), and the fourth was organisational antecedents for innovation (for example past experience with information technology projects, leadership and management capacity, effective data capture systems, slack resources). The fifth was organisational readiness for the SCR (for example, innovation-system fit, tension for change, power balances between supporters and opponents, baseline data quality). The sixth was the implementation process (including the nature of the change model and the extent to which new routines associated with the SCR aligned with existing organisational routines). The seventh influence was the nature and quality of links between different parts of the system, and the final one was the wider environment (especially the political context of the programme).

**Conclusion** Shared electronic records are not plug-in technologies. They are complex innovations that must be accepted by individual patients and staff and also embedded in organisational and inter-organisational routines. This process is heavily influenced at the micro-level by the material properties of the technology, individuals’ attitudes and concerns, and interpersonal influence; at the meso-level by organisational antecedents, readiness, and operational aspects of implementation; and at the macro-level by institutional and socio-political forces. A case study approach and multi-level theoretical analysis can illuminate how contextual factors shape, enable, and constrain new, technology supported models of patient care.

## Introduction

In 1998 the British prime minister, Tony Blair, expressed his vision for universally accessible electronic patients’ records: “If I live in Bradford and fall ill in Birmingham then I want the doctor treating me to have access to the information he needs.”^1^ This vision is linked at policy level with anticipated improvements in the efficiency, safety, equity, and cost effectiveness of care.^2^ ^3^ ^4^ It has not yet been realised on a large scale anywhere in the world, however, and many examples exist of it turning into an expensive failure.^5^ ^6^ ^7^

In general, the larger the scale of a new technology project in health care, the greater its chances of failure.^8^ ^9^ This is because healthcare information systems are complex; they raise unique technical, administrative, and security challenges; and introducing new technologies into a complex system requires extensive changes in individual roles, relationships, and business processes—the so-called “socio-technical” aspects of change. For this reason, the world waits with interest as the UK National Health Service unfolds its national programme for information technology, described as “the largest ever civilian IT project,”^10^ whose scope, scale, and value for money have been questioned by both policy makers and academics.^11^ ^12^ ^13^

The national programme for information technology is delivered centrally by Connecting for Health (part of the Department of Health) and locally by strategic health authorities and primary care trusts. One component of this programme is the summary care record (SCR), a centrally stored health summary created from a person’s general practice record. A person’s SCR will be accessible through a secured extranet (N3) by a wide range of NHS staff and is intended to support care when other records are unavailable (for example, in emergency and unscheduled care). People do not have to have an SCR, but if they do not want one they must actively opt out (box 1).

**Box 1 Key characteristics of the summary care record**

**Technology**

The summary care record (SCR) is a centrally stored summary of key medical details that is created from a person’s existing NHS record (currently, the detailed record held by their general practitioner) and made available to NHS staff in emergency and unscheduled care situations (emergency departments, general practitioner out of hours clinics, and walk-in centres). It is comparable to (but differs in important respects from) the emergency care summary in Scotland and the individual health record in Wales.

**What information does the SCR contain?**

Information held on the SCR is currently limited to current drugs, allergies, and adverse reactions (the “phase 1 upload”), but a minimum clinical dataset (for example, whether someone has diabetes) (the “phase 2 upload”) is being developed and added at selected sites.

**Security safeguards**

Extensive technical safeguards have been built into the SCR to prevent unauthorised access. Role based access controls restrict access to NHS staff with a legitimate relationship to the patient. Access by staff without such relationships are logged and audited; penalties for unauthorised access are severe and may include dismissal.

**Consent model**

At the time of the study, the consent model for the SCR was one of implied consent or “opt-out” (that is, unless a person explicitly withdrew consent, an SCR would be created), although this model is now being revisited. At the time of writing, patients may choose one of three options: “don’t store” (a blank SCR will be created; nothing will be uploaded beyond the demographic details that are already on the spine); “store and share” (a full SCR will be created); or “store but don’t share” (a full SCR will be created, but explicit consent must be obtained from the patient every time a health professional wishes to access it). An option for a “virtual sealed envelope” will also exist—a “store but don’t share” option applied to selective sensitive information.

In 2007-8, the SCR was introduced into several early adopter sites across the United Kingdom, of which this evaluation studied four. We sought to build a rich picture of the introduction, implementation, and routinisation of the SCR at these sites at both individual (patients, clinicians, managers) and organisational (general practice, primary care trust, unscheduled care settings, Connecting for Health) levels, so as to draw insights about the process of socio-technical change. The patients’ perspective has been reported separately.^14^

## Methods

### Management and governance

We set up a research advisory group with a lay chair and representatives of patients, clinicians, professional bodies (British Medical Association and Royal College of Nursing), and academic peers.

### Design and setting

We used mixed method case study evaluation across four SCR early adopter sites. Each site consisted of a primary care trust, participating general practices, and one or more linked unscheduled care setting (such as an emergency department, walk-in centre, out of hours service). Connecting for Health had selected these sites for inclusion in the early adopter programme in a competitive bidding process from 56 applicants on the basis of various criteria including quality of general practice data, track record of innovation with information and communications technology, and local software suppliers. The catchment populations of three sites (in the north of England and the Midlands) were of lower than average socioeconomic status and higher than average levels of limiting long term illness; the fourth site was an affluent rural area in the south of England with low levels of illness (see full report for demographic details^15^).

### Theoretical framework

The data collection was driven by Patton’s utilisation focused evaluation method.^16^ We used a multi-level theoretical framework of complex innovation in health service organisations that we had previously developed in a systematic literature review of some 600 sources (including several previous systematic reviews and meta-analyses),^17^ supplemented by more recent work on the role of information and communications technology innovations in organisational change efforts and the routinisation of complex innovations.^18^ ^19^ Box 2 summarises the components of the model. We adopted the stance that because of the complexity of the innovation, the dynamic and contingent nature of the implementation process, and the shifting environmental context (political, economic, technological), complex service level innovations are inherently unpredictable and that the search for reproducible “effect sizes,” “mediators,” and “moderators” is likely to prove fruitless. The best we can do is to explain what is happening as we observe it and reflect on it in a theory driven way. Our goal (which contrasts with that of many programme evaluators) was thus interpretation rather than prediction.^16^

**Box 2 Components of the diffusion of innovation model for complex innovations in health services^17^**

See text for definitions and examples of organisational components

**1. Material properties of the technology**

To be successfully and widely adopted, a technology must include key functionality and work smoothly and efficiently under real conditions of use

**2. Attributes of the technology as an innovation**

To be successfully and widely adopted, a technology must be seen by potential adopters as having

- Relative advantage (that is, clear benefits over existing technologies)
- Simplicity
- Compatibility with existing values and ways of working
- Trialability (can be tried out on a limited basis “without obligation”)
- Observability (benefits can be seen directly)
- Potential for reinvention (capacity for users to customise and adapt it)

**3. Concerns of potential adopters**

Adoption is a process, not a one-off event, and is influenced by concerns, including

- Before adoption—what are its properties and potential benefits?; what will it cost me?
- During early use—how do I make it work?; when and how should I use it?
- During established use—how can I alter or improve it?

**4. Communication and influence**

A person’s decision to adopt an innovation is influenced by

- Mass media (press, mail shots), which can raise awareness
- Interpersonal influence (by champions, opinion leaders, for example), which can change people’s attitudes towards adoption

**5. Organisational antecedents for innovation**

Organisations may be more or less innovative. Differences are explained by several factors:

- Absorptive capacity for new knowledge
- Leadership and management
- Risk taking climate
- Effective data capture systems
- Slack resources

**6. Organisational readiness for innovation**

An organisation must be “ready” for a specific innovation. Readiness includes

- Innovation-system fit
- Tension for change
- Balance between supporters and opponents
- Specific preparedness

**7. The implementation and routinisation process**

Implementing a complex innovation, and making sure it becomes business as usual, is a highly non-linear process, typically characterised by shocks and setbacks. Critical success factors include

- Appropriate change model (balance between “make it happen” and “let it emerge”)
- Good project management
- Autonomy of frontline teams
- Human resource factors, especially the selection, retention, continuity, and training of staff
- Alignment between new and old routines

**8. Linkage**

Innovation is more likely when there is

- Early and ongoing dialogue between the developers of the innovation, the change agents charged with promoting its adoption, and the end users
- Communication within the organisation and between similar organisations

**9. The wider environment**

Innovation in organisations is more likely when a “following policy wind,” a conducive socio-political climate, and specific incentives and mandates at national level are present

### Data sources and analysis

Data sources are summarised in box 3 and set out in detail in our full report.^15^ This study generated large amounts of qualitative data of different forms (such as field notes, documents, interviews, informal stories) as well as some quantitative data (such as closed item questionnaires, monitoring statistics). We processed (anonymised, indexed, and coded) all data and stored them securely. Analysis occurred in three overlapping stages: we analysed each data source separately by using an appropriate technique (for example, theory driven thematic content analysis for qualitative data); we further integrated these first order analyses by using narrative synthesis, so as to produce a coherent, multi-level interpretation of the story at each early adopter site; and we further synthesised insights from individual case studies in a cross-project analysis. We analysed data as soon as was practicable after we had collected them so as to feed emerging findings into the next phase of fieldwork. The synthesis phase involved interim presentations to participating primary care trusts and incorporation of their feedback on our interpretations.

**Box 3 Summary of data sources**

Full details of these data sources, including interview schedules, are given in the full report^15^

**Qualitative (non-numerical) data**

- 250 in-depth interviews with staff involved with the summary care record project or comparable projects, comprising
  1. 51 with Connecting for Health (CFH) staff
  2. 44 with primary care trust (PCT) staff
  3. 82 with staff in general practices (including some in non-participating practices)
  4. 41 with staff in unscheduled care settings
  5. 32 with staff and “expert patients” in voluntary sector, patients’ organisations, and other non-NHS stakeholder organisations (not including patient/carer interviews below)
- Approximately 1500 hours of ethnographic observation, comprising
  1. 400 hours at CFH (observing committees, working groups, team meetings, phone-ins, presentations, and informal contacts with CFH staff)
  2. 350 hours at PCTs (observing project board meetings, team meetings, engagement events, training events, and so on)
  3. 400 hours in general practices (observing reception areas, waiting rooms, and consultations)
  4. 150 hours in emergency and unscheduled care settings (observing reception areas, waiting rooms, consultations, and call centres)
  5. 40 hours in settings where a comparable innovation had been introduced (such as Scotland)
  6. 180 hours observing the voluntary sector, patients’ groups, campaigns, and so on
- Approximately 2500 pages of documentary data, comprising
  1. 1200 pages from CFH (committee and board papers, internal emails, internal and consultancy reports, contracts, press releases, and similar documents)
  2. 1300 pages from the early adopter PCTs and general practices (for example, project initiation document, board papers, minutes of meetings, progress reports to CFH, email exchanges)
- A small “mystery shopper” survey comprising 12 questions asked to advisers on the NHS Information Line, targeting issues that had emerged as confusing or controversial in our other data sources
- An analysis of the foreign language leaflets produced by CFH, in which each leaflet was shown to two bilingual people (one with a research/medical background and one selected to provide a “lay” perspective)
- Brief interviews with 103 patients or carers plus seven focus groups involving a total of 67 people who had potentially stigmatising conditions or difficulties accessing health care (reported in detail separately^14^)

**Quantitative (numerical) data**

- Publicly available data on population demographics, socioeconomic deprivation, burden of disease, and satisfaction with general practitioners’ services
- Closed item questionnaire items developed by our own team for interviews with staff in participating PCTs
- Regular uptake statistics produced by CFH and participating PCTs—for example, on the proportion of records uploaded by a given date
- The TNS UK tracker surveys of public attitudes to the national programme for information technology and SCR, summarised in detail in our full evaluation report^15^

## Main findings

### SCR early adopter programme: overview

Connecting for Health’s approach to the SCR programme was one of active control, characterised by detailed planning, tight monitoring, extensive documentation, and frequent reporting. This was to some extent mirrored at primary care trust level, where implementation had several formal stages: set-up (establish local infrastructure; recruit staff); preparation (ensure that practices meet minimum data quality standards, raise public awareness of the programme, and provide patients with an opportunity to opt out if they did not wish to have a record); “go live” (create SCRs from local general practice records, also referred to as “the upload”); and deployment (support the use of the SCR in emergency and unscheduled care settings).

The first early adopter site began preparation in spring 2007, and the first SCRs were created in June 2007; deployment began on a limited scale in October 2007. The second site followed soon after, but at the third and fourth sites (which used different general practice suppliers), go live was delayed by several months, mainly because of the failure of software contractors to deliver key technologies to contracted schedules, so no data were collected from emergency and unscheduled care settings at those sites. As of the end of April 2008, the SCRs of 153?188 patients at the first two sites had been created. A total of 614?052 patients in four early adopter sites had been sent a letter informing them of the programme and their choices for opting out of having an SCR (or limiting access to it). Of these, fewer than 1% had opted out of having an SCR and 0.03% had asked for data on their SCR not to be shared.

Many technical glitches and operational problems occurred with the SCR and the technical infrastructure that supports it. This is not surprising in a project of this scale and complexity, but even relatively minor problems sometimes led to long delays and considerable frustration in all participating organisations. This occurred in a context in which Connecting for Health was considered to be under pressure from government to redress a “worrying lack of progress” on the national programme for information technology.^20^ Non-participation of general practices in the programme ranged from 7% to 42% across the early adopter sites. This was due to a variety of reasons, including doubts about the benefits of the SCR, insufficient reimbursement, competing priorities, inadequate data quality, incompatibility of their software system, and ethical concerns. Key influences on how the programme unfolded are listed below.

### Material properties and attributes of the SCR

At the time of the study (May 2007 to April 2008), users perceived the SCR to be an immature technology, described as “clunky,” and to interface poorly with other information technology systems. Many people had given up using it “until it works better.” Wide variability existed among NHS staff on whether they felt the SCR had significant benefits, although most were broadly enthusiastic. A widespread perception existed that the consent model, the opt-out model, and role based access controls (box 1) were “too complicated to work in practice.” We noted an inherent imbalance between people who must work to upload patients’ SCRs (general practitioners and their staff) and those who will see its benefits most directly (staff working in emergency settings).

A small minority of general practitioners saw the SCR as fundamentally eroding the essence of their work and their professional identity. They felt strongly that their role was to “protect” their patients’ data rather than to “pass it to the government.” However, other general practitioners argued that contemporary health care requires a radical change in how confidentiality and privacy are defined (from a property of the individual doctor-patient relationship, mediated by the human qualities of the doctor, to a property of the system as a whole, mediated by technical and operational security measures).

### Concerns about the SCR and its use

The main concerns of general practices in early adopter sites were workload and the ethics of consent. Workload for phase 1 was lower than anticipated and mainly comprised the administrative task of meeting quality standards for data. General practitioners were concerned that an “opt-in” consent model for the imminent phase 2 upload (box 1) would generate a large workload for practices, especially for clinicians who may have to add information to records one by one. Other concerns included whether the implied consent model was legal, whether patients understood the choices they were being asked to make, whether the record was technically and operationally secure, whether participation in the programme would erode patients’ trust in the practice, and the risk of the system grinding to a halt during the upload. Concerns of staff in unscheduled care settings mainly related to the technical usability of the software.

### Influencing people’s decision to adopt the SCR

In common with previous research on the adoption of innovations by individual people,^21^ this study showed that mass media campaigns (mail shots, press coverage, road shows) were relatively ineffective in influencing people’s attitudes to the SCR.^14^ Connecting for Health recognised the need for interpersonal influence from the outset and appointed two “opinion leaders” (national clinical leads), who travelled the country to explain what the SCR was, hear the concerns of their fellow general practitioners, and try to make their audiences more receptive to the programme. These events were popular, and the national clinical leads were seen as credible, although many people thought that clinical engagement suffered through externally imposed timescales and milestones.

All participating primary care trusts had at least one local champion—an enthusiastic general practitioner or senior nurse who made a strong link between developing information technology infrastructure and improving care of patients,^22^ and who typically became a “super user” (a person showing early and sophisticated use of the SCR in a way that others could learn from^23^). These local champions typically held other roles in quality and professional development (such as trainer, appraiser, or member of the professional executive committee of the primary care trust) and framed the SCR programme as part of their wider quality role across the organisation. Sometimes “negative champions” opposed the programme. If these people occupied key positions (for example, on the local medical committee), they had significant influence on the pace of progress.

In general, general practitioners who also worked in the out of hours service were keen to see the SCR implemented. This is a classic “boundary spanning” phenomenon (in which people who worked in more than one organisation cross fertilised ideas between them). In common with the findings of previous studies,^17^ boundary spanners seemed to be powerful agents of change.

### Organisational antecedents for innovation

Several characteristics of participating organisations seemed to be important in explaining the fortunes of the SCR.

#### Absorptive capacity for new knowledge

Absorptive capacity is defined as a combination of formal expertise, informal organisational know-how, technical infrastructure, and relevant interpersonal networks.^24^ Practice 16, for example, was one of the first to successfully upload records, and it did so rapidly and efficiently. It had a top of the range information system, and the senior partner was technically keen and capable, with strong links to his system supplier and the national programme for information technology. Informatics training for staff was prioritised, in-house expertise in information technology was high, and technical know-how circulated informally among staff. In contrast, practice 37 experienced many delays and problems and was very dependent on external technical support. Despite the fact that the practice was large with an engaged management team, well appointed premises, and dedicated summarising staff, little in-house expertise in information technology existed. Indeed, this was not seen as a key skill needed by all staff but was delegated to the one “technical” general practitioner. Lack of relevant skills within the practice meant that little was learnt from encounters with technical support staff, and enthusiasm to develop internal networks to share technical learning was limited.

#### Leadership and management capacity

The importance of strong leadership, good strategic vision, good managerial relations, and committed and competent staff in introducing complex innovation is well established.^25^ In practice 1, some staff had initial reservations about the SCR project. One partner championed the project and persuaded others that it was well aligned with the overall goals of the practice and would benefit patients. The practice’s lead general practitioner for information technology subsequently took over responsibility for the project and also become a national clinical lead. In contrast, practice 6 signed up to the SCR project but was characterised from the outset by lack of leadership from the senior partner and to some extent also the practice manager; this practice needed considerable input from the primary care trust to achieve “go live.”

#### Risk taking climate

A risk taking climate is one in which experimentation is encouraged; failed projects lead to reflection and efforts to improve features of the system.^25^ For example, a general practitioner from practice 28 reflected, “We’ve gone through the years making mistakes, you see. And you learn from them. And that’s one of the things we can turn around and say, ‘We made the mistakes, we’ve learned from them and we have moved on’.” This general practitioner described several of what he called past “failures” in implementing information technology innovations, but the overall story was an impressive track record of success (for example, with Choose and Book, GP2GP, and ePrescribing).

#### Efficient data capture

Our findings confirmed that innovation is more readily introduced when systems are in place to capture data on performance and feed it into organisational learning.^25^ For example, practice 31, which easily achieved the data quality standards, showed a systematic and reflexive approach to data collection and analysis. The manager commented, “We find that for whatever reason something will come up [on an audit], and we ask, now is that a one-off or is it someone or something needing to change? It’s a training mechanism as well as an audit.” This approach contrasted markedly with that of practice 33, where errors detected through audit were given to the general practitioners to “fix” (with a view to meeting standards), but no reflection on, or efforts to correct, underlying system level errors occurred. The practice manager felt that “data quality is no better than it was before. . . [audit] has just created a lot of extra work.”

#### Slack resources

“Organisational slack” is a term used to denote spare time, money, or expertise that can be channelled into new projects.^26^ In this study, small practices in particular found that lack of any staff who could free up time to spend time on the project was an important barrier to successful implementation. Practice 33’s manager, for example, commented, “They initially sent someone out to explain what needed doing, left me a list to work on. . . [it] would have been nice to have money and staff to work on it. I think that bigger practices would find it easier to do; in larger practice with more staff some of the work could be delegated out, but in this practice there’s no one else to do it.”

### Organisational readiness for SCR

In addition to general antecedents, several factors specific to the SCR helped to explain the success of efforts to introduce it locally.

#### Innovation-system fit

Innovation-system fit refers to the degree of alignment between the organisation’s wider development goals and the introduction of a specific innovation.^17^ For example, the manager of out of hours clinic E linked the SCR to a wider strategy for an integrated service to replace the previous fragmented one. She talked about unacceptable experiences for patients (long waits, questionable clinical care, poor premises, loss of dignity) and financial waste (for example, through unnecessary acute admissions) and described a vision of a “state-of the art” out of hours service with a new building (currently under construction), well trained professional staff, efficient infrastructure, and seamless communication between organisations.

#### Tension for change

Tension for change refers to the extent to which people are uncomfortable with the status quo and feel that something has to change.^27^ In out of hours clinic E, for example, clinicians felt that assessing patients without access to records was “stabbing in the dark” and placed patient safety at risk. Considerable enthusiasm existed for the SCR as a potential solution to this unacceptable situation. In contrast, several practices that had initially signed up to the SCR project but had subsequently withdrawn from it reflected that little tension for change had existed within their organisation: “We were very, very strongly encouraged to go ahead [by the primary care trust]. [We] wouldn’t have done it otherwise” (manager from practice 14).

#### Balance of support

Where supporters of a complex innovation outnumber its opponents and are more strategically placed, innovation is generally more successful^27^; our data affirmed this. In practice 2, for example, one of the three partners and the practice manager strongly supported the SCR project and the senior partner was also broadly supportive. The third partner was somewhat opposed, but his position was not strong enough to stop the practice from participating. In practice 28, the general practitioner information technology lead was very enthusiastic about the SCR and many other staff were also keen. The primary care trust and Connecting for Health initially saw it as a “flagship” practice for the SCR programme. However, the *Caldicott Guardian* was strongly opposed to the implied consent model and provided well argued resistance to it. To the surprise and disappointment of the trust, practice 28 withdrew from the programme.

#### Specific preparedness

Innovation in organisations is more successful when preliminary groundwork has been done to the necessary standard.^17^ Many practices attributed the relatively smooth upload of records to the SCR to good overall quality of data, usually linked to the information management and technology directly enhanced service initiative (a financial incentive scheme to improve the quality of data). In contrast, one practice that struggled with preparations for the upload phase subsequently reflected, “We were surprised we were chosen [as an early adopter of the SCR], because we have only computerised 60% of our records so far, and when we started we were on nothing” (manager, practice 6).

### The implementation and routinisation process

Making the SCR “business as usual” in any organisation involved a lot of work. Several influences were evident.

#### Appropriateness of change model

All complex innovation requires a judicious balance between managerial (“make it happen”) and developmental (“let it emerge”) approaches.^17^ Widespread resentment existed among participating primary care trusts that Connecting for Health pushed forward on a tightly managed, largely non-negotiable timetable despite the immaturity of technical solutions. General practices and unscheduled care providers resented that they were in turn pushed excessively by primary care trusts. Some clinicians’ leaders believed that they were not adequately consulted because of spurious deadlines. Many interviewees complained that, in the circumstances, the unseemly haste was somewhat absurd and felt that depth of commitment had suffered.

#### Effective project management

Clear goals, realistic milestones, efficient delivery and coordination, and attention to follow-through are well established elements of the success of any complex project. In general, operational management was good in most early adopter organisations. When this was not the case (for example, when posts were unfilled, managers lacked key skills, or workload outstripped available funding for staff), organisations struggled and delays occurred: “We haven’t been given any resources to deal with the extra workload for returned mail [or] clinicians having to add to the records for phase 2” (manager, practice 21, which withdrew from the early adopter programme).

#### Autonomy of front line teams

Complex innovation is generally more successful if responsibility for operational decision making is devolved to front line teams.^17^ To some extent, our data affirmed this. Several practices, for example, described a process of adapting the instructions provided by Connecting for Health or the primary care trust so as to make them workable locally: “The process maps were just triangles, squares, and circles and didn’t mean anything, and something shorter and more concise was needed for in-house use. It’s my girls from [the practice] having to do this” (manager, practice 1). However, we also found that many practices were happy for the primary care trust to take over the change process and seemed to welcome the lack of autonomy: “[We have] hardly any personal responsibility” (manager, practice 22); “[We are] waiting for instructions” (manager, practice 17).

#### Human resource problems

Successful implementation generally depends on recruitment and retention of staff with key skills, knowledge, and credibility.^17^ One of the most successful practices (practice 2) had a longstanding and close knit team of staff—the two senior administrators had been there for more than 20 years—and a good balance of skills including information technology and project management. Staff attributed their success to the input, enthusiasm, and goodwill of all team members. At primary care trust level, limited success with implementation of the SCR was often attributed to failure to appoint staff with key experience and qualities (especially in relation to data quality work).

Another aspect of human resources is training—especially hands-on, on the job training for individual staff members; informal and “helpdesk” support for new users; and team training for tasks requiring teamwork.^17^ In practice 17, training was viewed particularly positively because the trainer had been “professional” and “flexible”—that is, he had run several sessions in order to cover all staff and had personalised the sessions to individual needs. Staff at this practice were confident and keen to start work with the new technology. More commonly, however, practices felt that training had been badly timed or difficult to apply in practice. The manager of practice 8 commented that “the PCT people arrived with their packs and slide shows, and it’s just nothing like that” and that after this initial training session “we were dreading it [the SCR upload].”

#### Alignment of routines

A key determinant of successful innovation is whether the new routine associated with the innovation aligns rather than conflicts with existing organisational and inter-organisational routines.^19^ In one participating emergency department (D), a well established “target chaser” role existed, which already involved logging on to a computer and bringing blood test results or other clinical information from the record to the attending clinician. In this setting, the SCR was described as “no more of a problem than any other aspect of medical records” (manager, site D). In another emergency department (A), the SCR could not be accessed as part of the receptionist’s role, because of the promise made in the Connecting for Health confidentiality leaflet that receptionists would not see patients’ clinical details. The task was allocated to healthcare assistants, but it was poorly aligned with their existing role, so accessing patients’ SCRs proved difficult operationally.

### Links between different parts of the system

One system level aspect of innovation previously shown to have a significant impact on innovation success is “linkage”—that is, ongoing formal and informal exchange of knowledge between different parts of the system, which tends to lead to a shared vision and common language for describing the innovation and to mutual understanding.^17^ In this study, we identified two important problems with linkage.

#### Linkage between technical developers and SCR users

Links between the technical developers of the SCR and its end users (such as general practitioners) tended to be characterised by lack of shared vision or language and low levels of mutual understanding. For example, one general practitioner who visited Connecting for Health’s technical base to talk to technical staff expressed surprise that “we were having to explain to them how it [the user interface of the SCR] worked rather than the other way round, yet until that point I thought they were the experts” (general practitioner, practice 2). Many end users attributed the SCR’s persistent “clunkiness” to poor linkage with the product’s designers.

#### Intra-organisational and inter-organisational knowledge sharing

The early adopter programme was characterised by relatively weak lateral links between participating organisations in relation to their work on the SCR. One primary care trust manager, for example, felt that “we couldn’t learn from X [neighbouring trust, also an early adopter site]; they’re very different from us and we had to work it out ourselves in a way that was relevant to us.” Connecting for Health held one or two networking events to bring people from different early adopter primary care trusts together. These events seem to be valued by participants: “Very useful—I’ve met people facing the same problems as I’m facing” (comment on evaluation form). We found few examples of specific exchange of knowledge, however. This may partly explain why some participants felt a sense of “reinventing the wheel” rather than building on the experience of others.

### Wider socio-political environment

The official policy wind from the Department of Health was certainly blowing in the direction of the SCR, but other forces were operating in the opposite direction. In particular, many stories in the media in 2007-8 reported large scale losses of data by government and the NHS; a strong civil liberties movement was arguing for less state control of private data, including opt-out campaigns led or endorsed by doctors; and some sectors of the medical press presented a series of small incidents which (allegedly) “proved” that the whole SCR initiative was a “fiasco.” All this contributed to a climate of uncertainty, which some practices cited as a reason for not wishing to be involved in the programme.

## Discussion

This case study, as well as our linked paper on the perspective of patients,^14^ has illustrated that shared electronic patients’ records are not plug-in technologies. They are complex innovations that must be accepted by individual patients and staff and embedded in organisational and system level routines. This process is heavily influenced at the micro-level by the material properties of the technology, people’s attitudes and concerns, and interpersonal influence; at the meso-level by organisational antecedents, readiness, and operational aspects of implementation; and at the macro-level by institutional and socio-political forces. The use of a case study approach based on extensive ethnography allowed us to document how interactions at these multiple levels shape, enable, and constrain the introduction of technology supported innovations in clinical care.^28^

### Self selecting innovators

All early adopter primary care trusts studied in this evaluation, and many participating general practices, scored highly on organisational antecedents for introducing new technologies, organisational readiness for the SCR, and operational aspects of managing the SCR project. These organisations had been selected for key characteristics (such as a track record of successful innovation in information technology) that accounted at least in part for their success and were also self selecting for “readiness” for the SCR. As the SCR programme expands, organisational weaknesses that were not seen in the early adopters are likely to become apparent. One way of tackling this is to try to build absorptive capacity through approaches such as the information technology training and facilitation for practices offered by the PRIMIS+ support team (see www.primis.nhs.uk/). Another is to continue to work proactively with both national and local opinion leaders and champions to ensure that the impact of interpersonal influence on clinical engagement (and hence on readiness of primary care trusts and general practices) is maximised.

### Consent model

One of the least popular aspects of the SCR programme was the “hybrid” consent model (box 1), which some participants viewed as unethical (because records are initially uploaded without explicit consent) and others as unworkable (because, in phase 2, clinicians will be required to obtain explicit consent from millions of patients). These positions represent the poles of a trade-off between high coverage of the population and gaining explicit consent for every record uploaded. Shared electronic record programmes in Scotland (emergency care summary), Wales (individual health record), and France (dossier médical personnel) have to some extent squared this circle by combining “implied consent to upload” with “explicit consent to view” at the point of care, although this has not been without controversy.^29^ ^30^ ^31^ ^32^ Connecting for Health is reviewing the SCR consent model in the light of our findings.

### Workload

Our suspicion that the SCR programme would be characterised by a high degree of uncertainty and unpredictability, and that the burden and impact of the programme would be impossible to quantify with any precision, was confirmed. Several stakeholders wanted this evaluation to “measure the workload” associated with the creation and deployment of the SCR. Although some people saw this as a simple exercise in accounting for time spent on SCR related tasks, the evaluation revealed a more fluid, unpredictable, and complex picture; the burden and impact of the SCR programme was impossible to quantify with any precision. For example, work on implementation of the SCR overlapped with other work (most obviously, the duty of all doctors to maintain accurate and up to date records) and increased substantially with the assiduousness of efforts to encourage patients to consider their choices.

### Technology push or socio-technical change?

The predominant change model adopted for the SCR programme was one of “technology push”—centrally driven, rationalistic, with a focus on documentation and reporting, and oriented to predefined, relatively inflexible goals. Connecting for Health has been criticised in the past for such an approach and is actively seeking to change it.^8^ When we began this evaluation in April 2007, for example, it was already something of a cliché within Connecting for Health that the national programme for information should be viewed as “10% technology, 90% business change,” although many staff equated the latter with the provision of business tools. Nevertheless, coexisting with Connecting for Health’s technology push model (and more prevalent in some sections of the programme than others) were occasional initiatives such as away-days, networking events, and consultations that resonated with more contemporary models of change built around theories of co-evolution and knowledge creation, and which reflect a “socio-technical pull” model.^15^ Our data, along with research on comparable initiatives within and outside the UK,^5^ ^6^ ^7^ ^33^ suggest that as the SCR programme expands, further movement in this socio-technical direction is likely to improve its chances of success.

A socio-technical change model would transform the SCR programme substantially—for example, the SCR would no longer be seen as an end in itself (with success measured in terms of number of records uploaded and extent of use) but as a means to other ends (with success being defined in terms of a range of locally relevant ends, for which the SCR would be provided as a resource). Whereas Connecting for Health staff talked about “implementing the SCR,” primary care trusts generally spoke about integrating the SCR as a part of the infrastructure for a wider strategic change. Emphasis would need to shift from project management (that is, running a circumscribed initiative with a predefined, measurable goal by working systematically and fixing problems along the way) to programme management (that is, bringing together multiple elements in iterative and adaptive ways against an ever changing background context, towards a more abstract goal such as “improving access to health records in emergency settings”). This goal needs to be continually redefined and negotiated throughout the innovation journey but is better able to transcend system shocks (such as shifts in technology specification or prevailing information technology policy).^34^ The skills of project management relate to planning, organising, monitoring, adjusting, documenting, and reporting. Those of programme management include negotiation, sense making, synthesis, and (above all) situational judgment. Both Connecting for Health and primary care trusts might consider selecting and developing staff for this last skill set.

### Time to reappraise Connecting for Health’s goals?

However, political expectations could stymie this radical shift in the change model. A widespread assumption is that the SCR programme should be evaluated primarily in terms of the extent to which it is on (or behind) schedule, rather than by softer, more emergent metrics such as the extent of clinical and public engagement or innovation-system fit. Although levelling criticism at Connecting for Health for using an out of date change model is easy, the programme is constrained by the fact that the various instruments of governance for the national programme for information technology (see, for example, reports from the National Audit Office^11^ and Public Accounts Committee^12^) have largely failed to recognise the incompatibility of a socio-technical approach to change with fixed, time limited goals and milestones.

### “Realising the benefits”—a one sided vision?

Much debate about the SCR programme is currently couched in the discourse of “benefits realisation” (for example, Connecting for Health and strategic health authorities are expected to develop and apply formal metrics to measure the number of lives saved, adverse reactions averted, duplicate tests avoided, or patients empowered per pound invested in the programme). This approach assumes not only that developing and applying meaningful metrics in this area is possible but also that such metrics can be established in advance and used to performance manage the public bodies charged with implementing the programme. Both assumptions are highly questionable. Berg, for example, has argued persuasively that any measures of the success of large scale information technology programmes in health care must be developed organically alongside the operational characteristics of the technology in use, through a process of technological (re)design, consultation, negotiation, and policy deliberation—and the fitness for purpose of such metrics must be continually questioned as the programme develops.^35^ In our view, for the SCR to have any chance of bucking the current trend of failed large scale information technology projects in health care, politicians, press, and public must begin to conduct their deliberations within this wider socio-technical discourse.

**What is already known on this topic?**

Many countries have recently introduced shared electronic patients’ records to support care in emergency and unscheduled settings and improve coordination of care
Information technology projects in health care are known to require social as well as technical change

**What this study adds**

The fortunes of the summary care record (SCR) programme so far are explained by properties of the technology itself, attitudes and concerns of users, organisational antecedents and readiness, and the wider socio-political context
Shifting the change model from “technology push” to “socio-technical development” may improve the chances that the SCR will be used extensively to support patient care in emergency and unscheduled care settings
An evaluation approach that aims for interpretation and understanding rather than prediction and “effect sizes” can generate important insights about the mechanisms of success or failure in complex change programmes

## Notes

We thank the research participants for their cooperation and support. The research was overseen by a multidisciplinary advisory group, which included patients’ representatives. Mike Pringle, Claudia Pagliari, Judith Lathlean, Kath Checkland, and Trish Groves gave helpful feedback on earlier drafts of this paper.

Ethical approval: Thames Valley Multicentre Research Ethics Committee (06/MRE12/81 and subsequent amendments).

Provenance and peer review: Not commissioned; externally peer reviewed.

## Notes

Cite this as: *BMJ* 2008;337:a1786
